# Long-Term Stability of Bacterial Associations in a Microcosm of *Ostreococcus tauri* (Chlorophyta, Mamiellophyceae)

**DOI:** 10.3389/fpls.2022.814386

**Published:** 2022-04-08

**Authors:** Sophie Vacant, L. Felipe Benites, Christophe Salmeron, Laurent Intertaglia, Manon Norest, Adrien Cadoudal, Frederic Sanchez, Carlos Caceres, Gwenael Piganeau

**Affiliations:** ^1^Integrative Biology of Marine Organisms (BIOM), Sorbonne University, Centre National de la Recherche Scientifique, Oceanological Observatory of Banyuls, Banyuls-sur-Mer, France; ^2^Sorbonne Université, Centre National de la Recherche Scientifique, Observatoire Océanologique de Banyuls, FR3724, Banyuls-sur-Mer, France

**Keywords:** picophytoplankton, mutualism, symbiosis, *Ostreococcus*, heterotrophic bacteria, vitamin B12, secretion systems

## Abstract

Phytoplankton–bacteria interactions rule over carbon fixation in the sunlit ocean, yet only a handful of phytoplanktonic–bacteria interactions have been experimentally characterized. In this study, we investigated the effect of three bacterial strains isolated from a long-term microcosm experiment with one *Ostreococcus* strain (Chlorophyta, Mamiellophyceae). We provided evidence that two *Roseovarius* strains (Alphaproteobacteria) had a beneficial effect on the long-term survival of the microalgae whereas one *Winogradskyella* strain (Flavobacteriia) led to the collapse of the microalga culture. Co-cultivation of the beneficial and the antagonistic strains also led to the loss of the microalga cells. Metagenomic analysis of the microcosm is consistent with vitamin B12 synthesis by the *Roseovarius* strains and unveiled two additional species affiliated to *Balneola* (Balneolia) and *Muricauda* (Flavobacteriia), which represent less than 4% of the reads, whereas *Roseovarius* and *Winogradskyella* recruit 57 and 39% of the reads, respectively. These results suggest that the low-frequency bacterial species may antagonize the algicidal effect of *Winogradskyella* in the microbiome of *Ostreococcus tauri* and thus stabilize the microalga persistence in the microcosm. Altogether, these results open novel perspectives into long-term stability of phytoplankton cultures.

## Introduction

Bacterial–phytoplankton interactions in the sunlit ocean fuel the biological carbon pump (Field et al., 1998) and are fundamental for our understanding of the base of the food web in marine ecosystems (Azam and Malfatti, 2007). The interactions between bacteria and phytoplankton are multifarious and may span the spectrum of relationships from mutualistic (Amin et al., 2015; Choix et al., 2018; Cooper et al., 2019) or opportunistic (Pinto et al., 2021) to antagonistic (Fukami et al., 1997; Mitsutani et al., 2001; Sohn et al., 2004; Wang et al., 2010). Mutualistic interactions are generally driven by reciprocal needs of both taxa specific bacteria and phytoplankton partners (Mönnich et al., 2020). These requirements encompass essential trace elements, nutrients (Amin et al., 2015), and vitamins, such as in the production and acquisition of the B vitamins (Cooper et al., 2019), given that many phytoplanktonic microalgae are confirmed auxotrophs for vitamin B_12_ (Croft et al., 2005). In turn, phytoplankton cell wall products and other exudates can be utilized as carbon sources to heterotrophic bacteria (Myklestad, 1995; Christie-Oleza et al., 2017). Consequently, the phytoplankton dynamics and biomass production (Suminto, and Hirayama, 1997) in the ocean (Buchan et al., 2014) are altogether affected by these range of interdomain interactions which still remain enigmatic and poorly studied.

Following isolation from environmental sampling, photosynthetic eukaryotes maintained in culture collection are usually sustaining a diverse microcosm of heterotrophic bacteria, which are expected to benefit from the extracellular products of the microalgae (Bell and Mitchell, 1972). The relative frequency of bacteria to microalgae is highly variable from as low to 1:100 (Bacteria:Microalgae) to 4:10 in healthy cultures (Abby et al., 2014a) and is likely to depend on several different factors. Among these factors, there is the identity of the microalga, since the phylogenetic spread of phytoplanktonic microalgae spans the entire eukaryotic tree of life (Not et al., 2012), the composition of the culture media, the physiological state of the microalgae, the physiological state of the bacteria, and the diversity of the bacterial community present. For example, the bacteria-to-microalgae ratio has been reported to vary with the age of the culture in the microalgae *Ostreococcus tauri* (Mamiellophyceae, Chlorophyta), a photosynthetic picoeukaryote which has been previously isolated from a Mediterranean lagoon (Courties et al., 1994) and the NW Mediterranean Sea (Grimsley et al., 2010). During exponential growth phase, the microalgae outnumber the bacteria, whereas the bacteria may outnumber the microalgae at a 50:1 ratio during the stationary phase and even more significantly so during the decay phase (Lupette et al., 2016). The advances in genome sequencing of phytoplanktonic eukaryotes has unraveled an unexpected genomic diversity of associated bacteria (Abby et al., 2014a; Rosana et al., 2016; Rambo et al., 2020). However, precise knowledge about the mutualistic, opportunistic, or antagonistic nature of the interaction and the estimation of the effect on microalgae growth or long-term stability requires co-cultivation of the microalgae and the bacterial partners (Amin et al., 2015; Behringer et al., 2018; Johansson et al., 2019; Lian et al., 2021; Pinto et al., 2021).

In our study, we took advantage of a microcosm containing *O. tauri* and a bacterial microbiome without external input, *pour ainsi dire* “in lockdown,” which had maintained the microalga for more than 1 year, to characterize the pairwise interaction between the microalga and the three bacteria isolated from this microcosm. Like many phytoplanktonic microalgae, *O. tauri* is auxotrophic for vitamin B_12_ as it requires vitamin B_12_ for growth and its genome does not encode the B12-independent form of methionine synthase (METE) (Helliwell et al., 2011). We first performed co-culture experiments to identify the nature of the short-term and long-term dynamics (up to 231 days) between the microalga and each individual bacterial strain as well as the dynamics between the microalga and the three combinations of bacterial strains. Second, we sequenced and analyzed the microcosm to investigate total bacterial diversity and the relative frequency of the different bacterial species present. We also investigated the genetic complementarity of the bacterial metagenome-assembled genomes (MAGs) for genes that may inform about the nature of the interaction with the microalgae: the genes involved for vitamin B12 synthesis and for the presence of bacterial secretion systems.

## Materials and Methods

### Phytoplankton and Bacterial Strain Isolation From the Microcosm

A microcosm experiment was started in triplicate with *O. tauri* RCC4221 100-ml cultures in L1 media in 200-ml closed flasks (Sarstedt T75 ref 83.3911) opened weekly for sampling. The microcosm, culture, and co-culture experiments were performed at 15 μmol m^–2^ s^–1^ with shaking (135 rpm) in 12:12 light–dark conditions at 15°C. After initial discoloration of the culture, as previously observed when *O. tauri* cultures are not reinoculated with fresh media (Lupette et al., 2016), the culture regained the typical green color of *O. tauri* cultures after 1 month. Following 1 year of sustained green coloration, the identity of the microalgae was checked with strain-specific primers (Grimsley et al., 2010) and the long-term stability of *O. tauri* RCC4221 was confirmed. The bacteria were isolated from the microcosm by streaking an aliquot of the culture on marine agar (MA) Petri dishes (Difco 2216) and incubated at 20°C in the dark. Three different single colonies among the most dominant morphotypes were picked and subcultured two times on MA plates until getting pure cultures. Then, each selected strain was transferred onto marine broth (MB) tube at 20°C, 100 rpm in the dark. After 72 h of growth, 3 ml of these cultures was used for cryopreservation in 5% dimethylsulfoxide or 35% glycerol, put into a −80°C freezer, and added to the Banyuls Bacterial Culture Collection (as BBCC2900, BBCC2901, and BBCC2902, hereafter B2900, B2901, and B2902). About 1 ml of this resting liquid culture was pelleted for DNA extraction and 16S rDNA sequencing.

Axenic *O. tauri* cultures were obtained by adding 1% antibiotics to cultures at 10^6^ cell concentration in L1 ASW media as previously described (Sanchez et al., 2019). To investigate the effect of the co-culture of *O. tauri* on bacterial growth, we compared the temporal dynamics of bacteria in co-cultures and in a media without *O. tauri*, hereafter coined exudate media. The media experiments were prepared as follows: exponentially growing cultures of *O. tauri* in L1 medium were filtrated through 0.02 μm to keep *O. tauri* exudates neither larger than 20 nm particular organic matter nor microalga or bacterial cells.

The co-culture experiments were performed in 10-ml glass tubes as follows: 0.6 ml of bacterial cultures (at a cell concentration between 10^8^ and 10^9^ cells ml^–1^) was added to 6 ml of axenic microalga culture (4 × 10^7^ cells ml^–1^) grown in L1ASW media.

### Cytometry Measurements

For flow cytometry counts of microalgae and free-living bacteria, 0.05 ml of culture was sampled, diluted at 1:10–1:10,000 and fixed for 15 min in the dark with a final concentration of electron microscopy–grade glutaraldehyde of 0.25% and Pluronic F-68 of 0.01% (Marie et al., 2014), flash-frozen in liquid nitrogen, and stored at −80°C until the analysis. Cell counts were performed with a BD FacsCanto II Flow Cytometry System [3-laser, 8-color (4-2-2), BD-Biosciences] equipped with a 20-mW 488-nm coherent sapphire solid-state blue laser. Accurate analyzed volumes and subsequent estimations of cell concentrations were calculated using Becton-Dickinson Trucount™ beads. Phytoplankton and bacterial cells were discriminated and enumerated according to their side scatter properties (SSC) for both and red fluorescence (>670 nm) due to chlorophyll pigments or green fluorescence due to SYBR Green I staining of the bacterial DNA [1:10,000 final concentration (Marie et al., 1997)], respectively. Data were acquired using DIVA software provided by BD Biosciences.

### Metagenomics of Microcosm and 16S rDNA Sequencing From Bacterial Isolates

DNA extraction and purification for 16S rDNA sequencing of B2900, B2901, and B2902 were carried out with the Wizard^®^ Genomic DNA Purification Kit (Promega) according to the manufacturer’s instructions. PCR and 16S rRNA gene sequencing were done as previously described (Fagervold et al., 2013) using the BIO2MAR platform facilities. Universal bacterial primers 27F and 1492R were used for PCR amplification. PCR products were cleaned up with AmpliClean Magnetic Bead PCR Clean-up Kit (NimaGen). Cleaned amplicons were sequenced with internal 907R primer using the BigDye Terminator v3.1 Cycle Sequencing Kit (Applied Biosystems) and cleaned up with D-Pure Dye Terminator Removal kit (NimaGen). The cycle sequencing products were loaded into an AB3130xl genetic analyzer (Life Technologies). Partial 16S rDNA sequences of these three strains were completed with metagenomic contigs and full-length 16S rDNA sequences were submitted to GenBank under accession numbers: OK396682, OK396683, OK396702, and OK396703.

**FIGURE 1:**
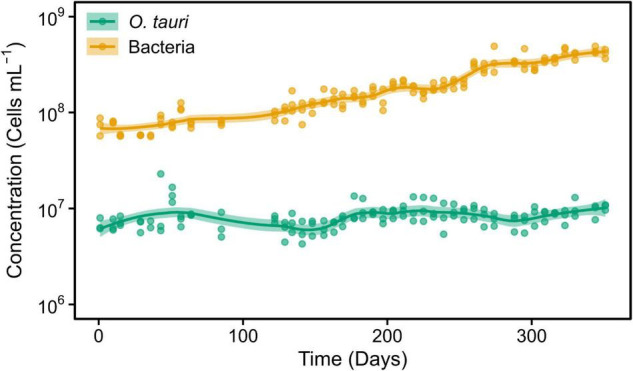
Concentrations of *Ostreococcus tauri* and bacteria during 50 weeks in the initial microcosm. Dots represent observed concentrations. Solid lines represent the temporal dynamics of the concentrations predicted by fitting local regression curves. Shaded areas represent the 95% confidence intervals (CIs). Note that a log_10_ scale is used in *y*-axis.

About 10 ml of the microcosm was sampled in February 2019 (between day 148 and 163 in Figure 1) and used for DNA extraction using a modified CTAB protocol (Winnepenninckx et al., 1993), concentrated to 0.043 ml (final concentration 0.03 mg ml^–1^), and sequenced with the Miseq Illumina technology (2 × 300 bp PE) on the bioenvironnement sequencing platform of the University of Perpignan (France). The 19.3 10^6^ PE reads were trimmed with TrimGalore with options –length 100 –paired,^1^ and the resulting 10.6 Gbp of DNA sequence was assembled with metaSPAdes version (Nurk et al., 2017) with parameters −k 55,77,99,127 meta. Scaffolds with 95% nucleotide identity over 1 kb BLASTN alignments with nuclear (Blanc-Mathieu et al., 2014) and chloroplastic or mitochondrial genomes (Blanc-Mathieu et al., 2013) of *O. tauri* were discarded from further analyses.

A total of two anchor datasets were built to screen the assembly. First, the reference dataset SILVA_138.1_ SSURef_NR99 (Quast et al., 2013) was used to identify 16S rDNA containing contigs, and the complete 16S rDNA sequence was annotated with RNAmmer (Lagesen et al., 2007). Second, the reference genes and corresponding amino acid sequences involved in the adenosylcobalamin (vitamin B12), and biotin and niacin pathways were compiled from Warren et al. (2002); Helliwell et al. (2016), and Cooper et al. (2019) and the Uniprot Knowledge Database (Boutet et al., 2007) and are listed in Supplementary Table 1. The presence or the absence of a gene was inferred from best BLASTN (16S rDNA) or TBLASTN (protein-coding genes) hit from the reference gene set onto the assembly with an *e*-value threshold <10^–5^.

The complete assemblies (available on 01/10/2021) of bacterial genomes that belong to the genera identified from the 16S rDNA were downloaded from GenBank: 86 *Roseovarius*, 75 *Winogradskyella*, 30 *Balneola*, and 68 *Muricauda*. Each contig from the metagenome was affiliated to the genus of the best blast hit (BBH) against this bacterial assemblies by BLASTN (*e*-value threshold < 10^–5^) (Altschul et al., 1990). The coverage of each contig was estimated by aligning the trimmed PE reads onto the assembly with BWA (bwa-mem2-2.0 version) (Li and Durbin, 2010) and SAMtools (Li et al., 2009). MAGs were obtained by binning contigs with BBH against assemblies of the same genus with similar coverage and GC content.

Each MAG was subsequently annotated with Prokka (Seemann, 2014). The predicted protein sequences were searched for secretion system components using the Macromolecular System Finder approach (Abby et al., 2014b) adapted for the detection of flagella and bacterial secretion system components in the TXSScan tool (Abby and Rocha, 2017) implemented on the Pasteur Institute Galaxy browser with default parameters.^2^

### Data Analysis

The dynamics of the microalgae and bacteria in the cultures were summarized by calculating the mean ± standard deviation (SD) of the minimum and maximum concentration of cells and their day of occurrence from the values obtained for each replicate. Moreover, we calculated the reproductive rate and the daily change in the concentration of microalgae (mean ± SD) between the maximum and the minimum concentration of cells and throughout the entire experiment for bacteria. In the case of the subculture of the initial microcosm, we calculated the initial local maximum concentration of microalgae instead of the global maximum. We compared the average values in each co-culture with those in the axenic culture of *O. tauri* with a *t*-test. In addition, to better appreciate the temporal dynamics of the microalgae and bacteria and to facilitate the visual comparison among treatments, we fitted local regression curves to the observations of concentration against time. To this end, we used the function geom_smooth of the R library ggplot2 (Wickham, 2011).

To analyze the effect of the bacteria on the temporal dynamics of the microalgae, we fitted segmented regression models within each culture type separately using segmented R library (Muggeo, 2008). We focused on the time interval comprised between the maximum and the minimum *O. tauri* concentrations. We considered the natural logarithm of the concentration of microalgae as response variable and time as predictor. In this way, (1) we were able to identify different temporal trends within the time interval analyzed and (2) the estimates of the slope had a biological meaning, as they corresponded to the intrinsic growth rate (*r*):


$$r=\frac{ln\left(\frac{N_{f}}{N_{i}}\right)}{t_{f}-t_{i}},$$

where *N*_*i*_ and *N*_*f*_ are cell concentrations at initial (*t*_*i*_) and final (*t*_*f*_) times, respectively. Then, we compared the breakpoint, i.e., the time at which the trend changed, and the slopes estimated for the axenic culture of the microalgae (control treatment) with those obtained for each co-culture of microalgae and bacteria (or combination of bacteria strains) by looking at the overlap of the 95% confidence intervals (CIs). We removed five observations of *O. tauri* concentrations because they corresponded to either (1) samples with zero flow cytometry counts that were followed by non-zero abundances or (2) samples with less than 10 counts preceded and followed by samples with zero counts. In the former case, concentrations were likely different from zero but no counts were detected, whereas in the latter case, cell counts very likely corresponded to flow cytometry noise. Anyway, the exclusion of these observations does not affect the interpretation of results.

All graphs and statistical analyses have been performed in R version 4.1.0 (R Core Team, 2021).

## Results

### *Ostreococcus tauri* Cultures Thrive in the Company of the Microbiome in the Microcosm

The *O. tauri* cultures inoculated in 200 ml L1 media and left without any external input maintained the typical light green coloration for 1 year. Subsequent sampling of this microcosm during 50 weeks (Figure 1) revealed a stable concentration of microalgae (C_*M*_) C_*M*_ = 10.40 × 10^6^ cells ml^–1^ and a slightly increasing concentration of bacteria (C_*B*_) up to C_*B*_ = 41.00 × 10^7^ cells ml^–1^, which corresponded to a 40:1 bacteria-to-microalgae ratio (Figure 1).

**FIGURE 2:**
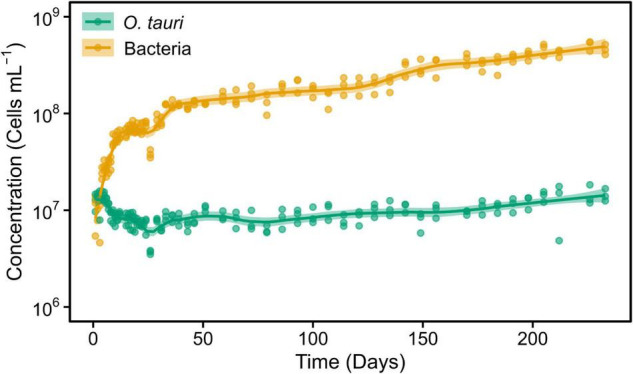
Concentrations of *O. tauri* and bacteria during 33 weeks in a subculture of the initial microcosm. Dots represent observed concentrations. Solid lines represent the temporal dynamics of the concentrations predicted by fitting local regression curves. Shaded areas represent the 95% CIs. Note that a log_10_ scale is used in *y*-axis.

To preserve the initial microcosm to proceed to a long-term monitoring, we decided to replicate the microcosm by subculturing 1 ml into tubes containing 3 ml of sterile L1 ASW media. This resulted in a change of the microalgae–bacteria dynamic and equilibrium (Figure 2). The concentration of the microalgae reached C_*M*_ = 1.04 × 10^7^ cells ml^–1^ within 2 weeks, whereas the bacteria reached the value observed in flasks after 22 weeks. However, and contrary to what had been observed in the original microcosm, there was a slight increase in the concentration of the microalgae after day 79 (reproductive rate = 1.01 ± 0.00, corresponding to 0.05 ± 0.02 × 10^6^ cells ml^–1^ day^–1^) and bacteria during the entire experiment (reproductive rate = 1.02 ± 0.00, corresponding to 1.94 ± 0.24 × 10^6^ cells ml^–1^ day^–1^). As a result, the bacteria-to-microalgae ratio ranged from 7:10 to 58:1 along this experiment. In conclusion, while the stability of the microalgae concentration observed in the original microcosm could not be strictly reproduced in the subcultures as the bacterial/microalgae ratio increased from 40:1 to 58:1, both the microalga and the bacteria could be maintained at high concentrations over the complete 231 days of the experiment (Figure 2).

### Some Bacteria Have Beneficial Effects Whereas Other Have Deleterious Effects on the Persistence of the Microalgae

**FIGURE 3:**
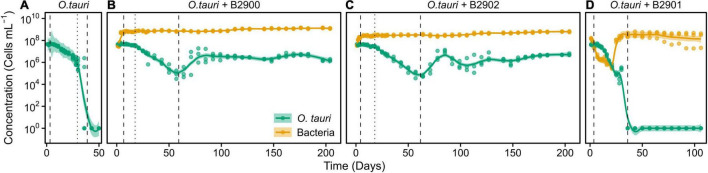
Concentration of *O. tauri* and different strains of bacteria in co-culture. **(A)** Axenic culture of *O. tauri*. **(B)** Co-culture of *O. tauri* and *Roseovarius* strain B2900. **(C)** Co-culture of *O. tauri* and *Roseovarius* strain B2902. **(D)** Co-culture of *O. tauri* and *Winogradskyella* strain B2901. Dots represent observed concentrations. Solid lines represent the temporal dynamics of the concentrations predicted by fitting local regression models. Shaded areas represent the 95% CIs of model fittings. Vertical dashed lines indicate the time of minimum and maximum average concentrations of *O. tauri*. Vertical dotted lines indicate the breakpoints of the segmental regression analyses detailed in Supplementary Figure 1. Note that a log_10_ scale is used in *y*-axis.

To assess the role of individual bacterial strains of the bacterial community of the microcosm, we isolated three strains and proceeded to co-culture experiments with *O. tauri* cultures treated with antibiotics. Long-term removal of 100% of the bacteria in an *Ostreococcus* culture below 10^4^ cells ml^–1^ is delicate to achieve, and this is likely to be due to bacterial persistence and the evolution of resistance to the antibiotics used. We therefore considered the *O. tauri* culture to be axenic as long as the bacterial signal detected from the cytometer was smaller than 1% of the signal of the microalgae throughout the first week after starting the culture. The axenic culture of the microalga collapsed after 39 ± 4 days (Figure 3A and Table 1), whereas the co-culture of the microalgae with either of the two *Roseovarius* strains did not collapse and followed similar dynamics (Figures 3B,C and Table 1): an initial 2–3 days stable concentration of the microalgae, followed by a decrease to a minimum C_*M*_ = 0.05 ± 0.03 × 10^6^ cells ml^–1^ (B2900, day 59 ± 4) and C_*M*_ = 0.05 ± 0.01 × 10^6^ cells ml^–1^ (B2902, day 62 ± 4), and a subsequent increase to reach concentrations that oscillated between 0.64 ± 0.61 × 10^6^ cells ml^–1^ (B2902, day 103 ± 4) and 14.86 ± 5.18 × 10^6^ cells ml^–1^ (B2900, day 78 ± 7). The concentration of bacteria increased at a mean reproductive rate = 1.02 ± 0.00 (B2900, 5.87 ± 0.47 × 10^6^ cells ml^–1^ day^–1^) and 1.01 ± 0.00 (B2902, 2.86 ± 0.34 × 10^6^ cells ml^–1^ day^–1^) and reached maximum C_*B*_ = 1393.33 ± 90.74 × 10^6^ cells ml^–1^ (B2900, day 183 ± 12) and 660 ± 36.37 × 10^6^ cells ml^–1^ (B2902, day 190 ± 12) (Table 2).

**TABLE 1:** Summary statistics (mean ± SD) of the dynamics of *Ostreococcus tauri* in the different culture treatments.

Culture	Min C_*M*_	Max C_*M*_	Day min C_*M*_	Day max C_*M*_	R_*M max*–*min*_	Δ C_*M max*–*min*_
Subcultures of initial microcosm	3.68 ± 0.17	14.50 ± 1.21	26.00 ± 0.00	3.66 ± 1.15	0.94 ± 0.00	−0.48 ± 0.05
*O. tauri* axenic	0.00 ± 0.00	50.24 ± 1.81	38.80 ± 3.83	3.20 ± 0.45	0.61 ± 0.03	−1.42 ± 0.13
*O. tauri* + B2900	0.05 ± 0.03	51.51 ± 5.04	59.33 ± 4.04**	6.67 ± 2.52	0.88 ± 0.01**	−0.98 ± 0.08**
*O. tauri* + B2902	0.05 ± 0.01*	46.61 ± 1.03*	61.67 ± 4.04**	4.33 ± 2.31	0.89 ± 0.02**	−0.82 ± 0.08**
*O. tauri* + B2901	0.00 ± 0.00	47.33 ± 4.00	36.00 ± 0.00	3.67 ± 3.06	0.58 ± 0.03	−1.48 ± 0.27
*O. tauri* + B2900 + B2902	0.04 ± 0.03	49.06 ± 1.53	68.70 ± 10.70*	6.00 ± 1.73	0.89 ± 0.03**	−0.80 ± 0.18*
*O. tauri* + B2900 + B2901	0.00 ± 0.00	46.08 ± 6.00	36.00 ± 0.00	5.00 ± 3.46	0.56 ± 0.04	−1.51 ± 0.32
*O. tauri* + B2901 + B2902	0.00 ± 0.00	45.10 ± 2.44*	36.00 ± 0.00	4.67 ± 2.08	0.57 ± 0.02	−1.45 ± 0.17
*O. tauri* + B2900 + B2901 + B2902	0.00 ± 0.00	44.99 ± 2.45*	36.00 ± 0.00	3.67 ± 3.06	0.58 ± 0.03	−1.40 ± 0.22

*Min C_M_: minimum concentration of microalgae (10^6^ cells ml^–1^). Max C_M_: maximum concentration of microalgae (10^6^ cells ml^–1^). Day min C_M_: day of the minimum concentration of microalgae. Day max C_M_: day of the maximum concentration of microalgae. R_M max–min_: reproductive rate between the initial maximum and the minimum concentration of microalgae. Δ C_M max–min_: average daily change in the concentration of microalgae between its maximum and minimum concentration (10^6^ cells ml^–1^ day^–1^). Significant differences with the axenic microalgal culture are indicated with one (p-value < 0.05) or two (p-value < 0.01) asterisks.*

**TABLE 2:** Summary statistics (mean ± SD) of the bacterial dynamics in the different culture conditions.

Culture	Min C_*B*_	Max C_*B*_	Day min C_*B*_	Day max C_*B*_	R_*B*_	Δ C_*B*_
Subcultures of initial microcosm	9.02 ± 3.93	511.00 ± 60.75	2.33 ± 0.58	226.00 ± 0.00	1.02 ± 0.00	1.94 ± 0.24
*O. tauri* + B2900	28.64 ± 2.27	1393.33 ± 90.74	2.00 ± 0.00	183.00 ± 12.12	1.02 ± 0.00	5.88 ± 0.47
*O. tauri* + B2902	34.11 ± 1.55	660.00 ± 36.37	1.00 ± 0.00	190.00 ± 12.12	1.01 ± 0.00	2.86 ± 0.34
*O. tauri* + B2901	0.32 ± 0.08	533.24 ± 111.69	21.00 ± 0.00	59.33 ± 14.57	1.00 ± 0.02	0.94 ± 1.90
*O. tauri* + B2900 + B2902	34.26 ± 1.04	582.33 ± 52.05	1.00 ± 0.00	194.67 ± 16.17	1.01 ± 0.00	2.58 ± 0.33
*O. tauri* + B2900 + B2901	14.90 ± 2.82	356.94 ± 41.87	7.00 ± 0.00	57.00 ± 12.12	1.01 ± 0.00	0.91 ± 0.53
*O. tauri* + B2901 + B2902	90.84 ± 3.99	1222.58 ± 51.38	3.67 ± 0.58	43.00 ± 0.00	1.01 ± 0.00	2.62 ± 0.11
*O. tauri* + B2900 + B2901 + B2902	55.29 ± 3.41	1149.38 ± 29.83	3.67 ± 0.58	36.33 ± 6.51	1.02 ± 0.00	2.93 ± 0.18
Exudate media + 2900	4.44	39.68	2.0	30	1.04	0.50
Exudate media + 2902	7.10	70.26	1.0	38	1.04	1.01
Exudate media + 2901	0.06	85.49	1.0	35	1.14	1.37

*Min C_B_: minimum concentration of bacteria (10^6^ cells ml^–1^). Max C_B_: maximum concentration of bacteria (10^6^ cells ml^–1^). Day min C_B_: day of the minimum concentration of bacteria. Day max C_B_: day of the maximum concentration of bacteria. R_B_: reproductive rate of bacteria throughout the entire experiment. Δ C_B_: Average daily change in the concentration of bacteria throughout the entire experiment (10^6^ cells ml^–1^ day^–1^). SD is not provided as there was only one replicate.*

In sharp contrast to the co-culture with *Roseovarius*, the co-culture of *O. tauri* and *Winogradskyella* strain B2901 leads to the loss of the microalgae population after 36 days (Figure 3D and Table 1). The decrease of *O. tauri* in the co-culture with *Winogradskyella* was even faster than the decrease observed in *O. tauri* axenic cultures before day 29, as the slope coefficient for the relationship between cell concentration and time is 24% lower and the 95% CIs of the slopes do not overlap (Table 3 and Supplementary Figure 1).

**TABLE 3:** Comparisons of the relationship between cell concentration and time for the axenic culture of *O. tauri* and co-cultures of *O. tauri* with different strains of bacteria from segmented regression analysis.

Co-culture	Time breakpoint	slope_1_	slope_2_	*R*_1_	*R*_2_
*O. tauri*	29.28 [27.17, 31.40]	−0.15 [−0.23, −0.07]	−1.30 [−1.74, −0.86]	0.86 [0.80, 0.93]	0.27 [0.18, 0.42]
*O. tauri* − B2900	17.56 [12.26, 22.85]*	−0.03 [−0.11, 0.05]	−0.15 [−0.16, −0.13]*	0.97 [0.90, 1.05]	0.86 [0.85, 0.87]*
*O. tauri* − B2902	18.02 [14.86, 21.18]*	−0.04 [−0.08, −0.01]	−0.15 [−0.16, −0.14]*	0.96 [0.92, 0.99]	0.86 [0.85, 0.87]*
*O. tauri* − B2901	No breakpoint	−0.44 [−0.52, −0.35]*	NA	0.65 [0.60, 0.70]*	NA

*The subindex (1 or 2) indicates whether the estimate corresponds to the time interval before (1) or after (2) the breakpoint. Intrinsic reproductive rates (R_1_ and R_2_) were estimated by the antilogarithm of the slopes. Confidence intervals of 95% are shown within brackets. Asterisk indicates that the 95% confidence interval does not overlap with the confidence interval estimated for axenic O. tauri.*

### Effect of the Microalga on the *Bacteria*

**FIGURE 4:**
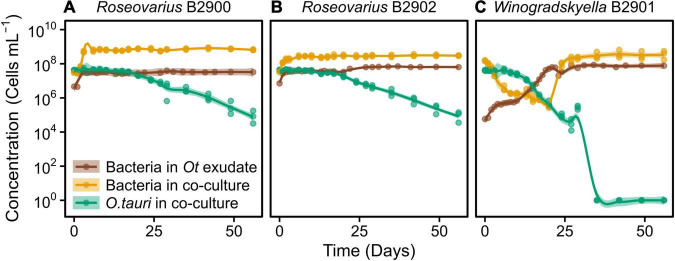
Concentration of different strains of heterotrophic bacteria growing alone in exudate media and co-cultured with *O. tauri*. **(A)** *Roseovarius* strain B2900. **(B)** *Roseovarius* strain B2902. **(C)** *Winogradskyella* strain B2901. Concentrations of *O. tauri* in co-cultures are also represented. Dots represent observed concentrations. Solid lines represent the temporal dynamics of the concentrations predicted by fitting local regression models. Shaded areas show the 95% CIs of model fittings. Note that a log_10_ scale is used in *y*-axis.

We further investigated the effect of the microalga on the bacteria by comparing the dynamics of the bacteria with and without (exudate media) the microalgae. For the two *Roseovarius* strains, co-culture and culture in exudate media led to initial growth (Figures 4A,B). For *Winogradskyella*, as opposed to culture in exudate media, co-culture led to a decay in bacterial concentrations until day 20 (Figure 4C), at which point the microalga decayed below 10^6^ cells ml^–1^ (Figure 4C). After day 20, the concentration of *Winogradskyella* increased to reach a plateau once the microalgae have died. As a conclusion, the microalgae and its exudate promoted the growth of *Roseovarius*, whereas the microalgae had a negative effect on the growth of *Winogradskyella*. Altogether, these observations suggest that the *Roseovarius*–*O. tauri* interactions are mutualistic and that the *Winogradskyella*–*O. tauri* interactions are antagonistic.

### Combining Antagonistic and Mutualistic Bacteria Does Not Reestablish Long-Term Survival of Microalga

**FIGURE 5:**
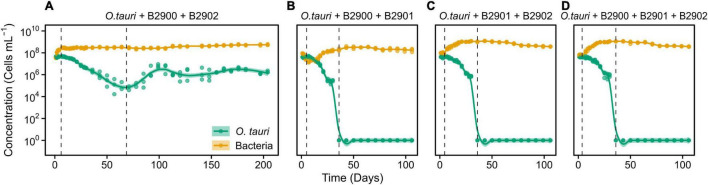
Dynamics of *O. tauri* concentration with different combinations of the bacterial strains in co-cultures. **(A)** Co-culture of *O. tauri* and the two *Roseovarius* strains. **(B)** Co-culture of *O. tauri*, the B2900 *Roseovarius*, and the B2901 *Winogradskyella* strain. **(C)** Co-culture of *O. tauri*, the B2902 *Roseovarius* strain, and the B2901 *Winogradskyella* strain. **(D)** Co-culture of *O. tauri*, with all three bacterial strains. Dots represent observed concentrations. Solid lines represent the temporal dynamics of the concentrations predicted by fitting local regression models. Shaded areas represent the 95% CIs of model fittings. Note that a log_10_ scale is used in *y*-axis.

We further investigated whether the antagonistic effect of the *Winogradskyella* strain could be compensated by the addition of the beneficial *Roseovarius* strains. This was not the case as, whenever the *Winogradskyella* strain was added into a co-culture experiment, the concentration of the microalgae would reach null values within 36 days (Table 1 and Figure 5). As a conclusion, the long-term stability of the microalgae in the microcosm experiment cannot be reproduced with the three isolated strains but with either one or the combination of the two *Roseovarius* B2900 or 2902 strains. Therefore, it is likely that additional bacteria are tempering with the antagonistic effect of *Winogradskyella* present in the microcosm.

### Metagenomic Insights Into the Total Bacterial Diversity Within the Microcosm

The assembly of the microcosm led to 1324 contigs (total 58.8 Mbp). Following the removal of the contigs aligning to *O. tauri* nuclear or organellar genomes (refer to section “Materials and Methods”), the bacterial diversity of the microbiome was inferred from 678 contigs (total 16.5 Mbp, average contigs length: 24.3 kbp, 240 contigs with length >1 kbp adding up to 16.3 Mbp). Screening this assembly for 16S rDNA confirmed the presence of *Roseovarius* and *Winogradskyella* sequences, which were 100% identical to the partial 16S rDNA Sanger sequencing performed on the bacterial isolates B2900, B2901, and B2902. The complete 16S rDNA of *Roseovarius* and *Winogradskyella* was extracted from the metagenome assembly and also the 16S rDNA sequence of two additional lineages: *Muricauda* and *Balneola*. Interestingly, and without surprise, the BBH of these 16S rDNA sequences against GenBank has all been sampled from the marine environment, which includes a strain isolated from the culture of a diatom microalgae (Table 4).

**TABLE 4:** Description of the four complete 16S rDNA sequences extracted from the metagenome.

16S rDNA accession	Length (bp)	BBH* (accession)	Identities	Origin of BBH
OK396682	1456	*Roseovarius mucosus* strain SMR3 (CP020474.1)	1455/1456	Isolated from a culture of the Diatom *Skeletonema marinoi*
OK396703	1512	*Muricauda marina* (NR_157633)	1481/1482	Isolated from marine snow of Yellow Sea (Su et al., 2017)
OK396683	1514	*Winogradskyella exilis* (NR_116736)	1448/1514	Isolated from a starfish (Ivanova et al., 2010)
OK396702	1523	*Balneola vulgaris* (NR_042991)	1371/1474	Isolated from the North-Western Mediterranean Sea (Agogué et al., 2005)

BBH, best blast hit against GenBank. *BBH from uncultured isolates has been excluded. Relative concentration of these four bacteria exists in the microbiome here.

**FIGURE 6:**
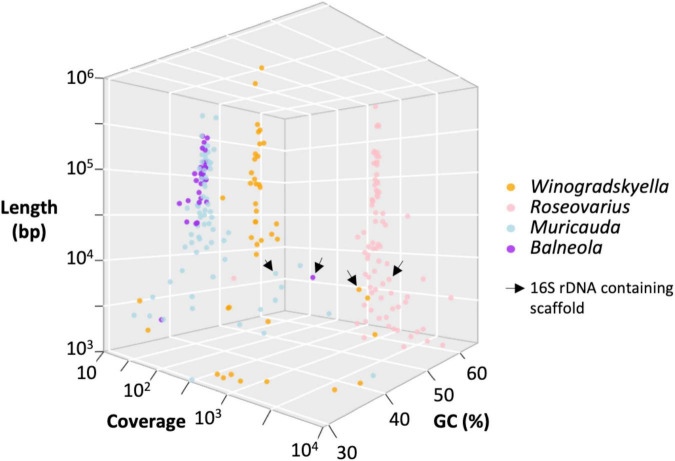
Distribution of the 240 sequences assembled from *O. tauri*’s associated bacterial community as a function of the contig length, GC content, contig coverage, and taxonomic affiliation inferred from taxonomic affiliation of best blast hit by BLASTN.

Taxonomic affiliation of the metagenome onto available assemblies assigned to these four bacterial genera led to 3.1 (*Winogradskyella*) to 4.8 Mb (*Muricauda*) MAG assemblies (Table 5). The MAG coverage and GC content statistics clearly separated *Roseovarius* (60% GC) and *Winogradskyella* (35% GC) affiliated contigs to the *Muricauda* + *Balneola* cluster (Figure 6). The *Roseovarius* MAG assembly shared a very high sequence identity (>99.9% nucleotide identity over >500 kbp) with a genome assembly affiliated to *R. mucosus* strain 85A, which has been isolated from the culture of a diatom microalgae, whereas the MAGs affiliated to *Winogradskyella*, *Balneola*, and *Muricauda* shared up to 86% nucleotide identity with sequences available from GenBank (Table 5). The percent of reads affiliated to each genera is thus 57% to *Roseovarius*, 39% to *Winogradskyella*, 1% to *Balneola*, and 2% to *Muricauda* (Table 5). The relative coverage of each MAG can, in turn, be used to estimate the relative frequency of each strain, that is, 49% of *Winogradskyella*, 47% of *Rosevarius*, 2% of *Muricauda*, and 1% of *Balenola*.

**TABLE 5:** Description of the four MAGs assembled from the microcosm.

	*Roseovarius* MAG	*Winogradskyella* MAG	*Balneola* MAG	*Muricauda* MAG
Total length (Mb)	4.7	3.1	3.2	4.8
Nb of contigs	79	43	32	71
GC (%)	60.3	35.2	41.6	41.6
Coverage	845.1	884.8	29.7	37.8
BBH accession	JAHXRP010000002.1	CP019388.1	LXYG01000014.1	JAFLNE010000001.1
BBH length (Mb)	0.58	3.3	0.32	1.1
Total length of BBH assembly (Mb)	4.8 (215 contigs)	3.3 (1 complete genome)	3.6 (20 contigs)	4.3 (20 contigs)
BBH name	*Roseovarius mucosus*	*Winogradskyella* sp.	*Balneola* sp.	*Muricauda* sp.
BBH origin	Culture of *Seminavis robusta* strain 85A (unpublished)	Seawater (unpublished)	Isolated from a culture of *Emiliania huxleyi* (Rosana et al., 2016)	Seawater (unpublished)
Maximum alignment/contig length (kb)	528/677	698/695	11/272	168/208
Identity over maximum aln (%)	99.98	86.2	85.2	85.5

*BBH, best blast hit by BLASTN.*

### Metagenomic Insights Into the Identity of the Vitamin B12 Producer and the Presence of Secretion Systems

The search for genes encoding for the niacin, biotin, and adenosylcobalamin pathways suggests the presence of a complete adenosylcobalamin (vitamin B12) pathway in the *Roseovarius* MAG with 18 genes detected (*cobA*, *cobI*, *cobJ*, *cobM*, *cobF*, *cobK*, *cobL*, *cobH*, *cobB*, *cobO*, *cobQ*, *cobU*, *cobP*, *cobD*, *cobS*, *cobV*, *CobC*, and *cobT*, Supplementary Table 2). As for the niacin and biotin pathways, which have been demonstrated to be incomplete from a *Dinoroseobacter* strain depending on *O. tauri* for niacin and biotin synthesis (Cooper et al., 2019), none of the MAGs seem to contain the complete gene complement for both pathways. The complete gene pathway for biotin has been identified in the *Muricauda* MAG, whereas it is incomplete in the *Roseovarius*, *Balenola*, and *Winogradskyella* MAGs (Supplementary Table 2). However, MAGs may not correspond to complete genome assemblies, so that the absence of a gene is not as informative as its absence from a complete genome assembly. Interestingly, available genome data from other strains suggest that the biotin pathway is complete in some *Roseovarius* and *Balneola* strains, that the niacin pathway is complete for some *Muricauda* strains, and that the vitamin B12 pathway is complete in some *Roseovarius* strains (Supplementary Table 1). As a conclusion, gene content analysis of the MAGs suggested that the *Roseovarius* strains present in the microcosm provide the microalgae *O. tauri* with vitamin B12.

Protein secretion systems are complex molecular machineries that translocate proteins through the outer bacterial membrane and sometimes through the membrane of an eukaryotic cell (Denise et al., 2020). The screening of the four MAGs for secretion systems (Abby and Rocha, 2017) did not allow the identification of the T4SS candidate gene complement within the MAGs. However, we identified the candidate genes for T1SS in all four MAGs, for T9SS in the *Winogradskyella* and *Muricauda* MAGs, and the candidate genes involved in the flagella within the *Roseovarius* MAG (Supplementary Table 3). We thus conclude that the *Roseovarius* strain may be motile, as observed in many Rhodobacteraceae (Bartling et al., 2018), though additional gene expression analyses would be required to check whether these genes are indeed expressed within the microcosm.

## Discussion

### Of the Importance of Long-Term Co-culture Experiments

We have isolated novel bacterial strains from a stable microcosm experiment started with a non-axenic *O. tauri* culture and provided evidence of the individual effects of these isolates on the microalgal growth and on long-term stability. The two *Roseovarius* isolates can be considered to be from the same species, as they share an identical 16S rDNA sequence, and the co-culture experiments demonstrated that they have a beneficial effect on the microalgal long-term survival. Analysis of the gene content of the *Roseovarius* MAG from the microcosm suggests that the *Roseovarius* strains are the unique producers of vitamin B12 in the microcosm, whereas *O. tauri* may provide niacin. However, there is no evidence of a type four secretion system (T4SS), whereas T4SS have been recently demonstrated as required for establishing a beneficial effect of another Rhodobacterales, *Dinoroseobacter*, on the growth rate of a dinoflagellate (Mansky et al., 2022). Unlike *Roseovarius*, *Winogradskyella* has a deleterious effect on the microalgal growth and long-term survival, which accelerates the decrease in the concentration of microalgae by 24% during the first 29 days of the co-culture (*R* = 0.65 vs. 0.86, for co-culture vs. axenic conditions, respectively; Table 3 and Supplementary Figure 1). The analyses of the gene content of the *Winogradskyella* MAG suggested that it encodes a T9SS, which provides either a means of movement called gliding motility or a weapon for pathogenic bacteria (Lasica et al., 2017). This complex has so far only been identified within the Bacteroidetes phylum (Abby and Rocha, 2017) to which *Balnoela*, *Muricauda*, and *Winogradskyella* belong to.

To our knowledge, phytoplankton–bacteria co-culture experiments are only exceptionally monitored for more than 30 consecutive days, with the notable exception of a 200 days *Synechococcus*–*Roseobacter* co-culture experiment (Christie-Oleza et al., 2017). Our study demonstrates the importance of long-term experiments as the first 15 days of co-culture may wrongly suggest stable concentrations of microalgae. Indeed, the evidence of the collapse of the microalgae populations in co-culture with both *Roseovarius* and *Winogradskyella* could only be observed after 15 days (Figure 5).

Obviously, the microalgal and bacterial cells will accumulate mutations and evolve over the course of long-term experiment (Krasovec et al., 2017). Interestingly, we observed that the number of bacterial cells tended to increase (slightly) over the course of the experiment (Figures 1, 2), whereas the number of microalgae only increased in the subcultured microcosm (Figure 2). Given that there is no external nutrient input into the system, this tendency suggests ongoing adaptation to the available resources in the microcosm.

The ratio of heterotrophic bacteria to microalgae at the end of both the initial microcosm (40:1) and the subculture of the microcosm (58:1) may be compared with the fraction of the photosynthetic pico-eukaryotic vs. heterotrophic bacteria fraction in the natural environment. This ratio can be estimated by cytometry and has been estimated to vary between 9:1 and 216:1 at the Station d’Observation Laboratoire Arago (SOLA, 42°29′N, 03°08′E) throughout the sampling performed during 2019 every 2 weeks (David Pecqueur, personal communication). Nevertheless, absolute concentrations in our experiments were markedly higher than in SOLA (bacteria range = 0.08 × 10^6^–0.22 × 10^6^ cells ml^–1^; picoeukaryotes range = 0.49–13.90 × 10^3^ cells ml^–1^), and this is likely the consequence of the initial higher availability of nutrients in the L1 culture media when the microcosm experiment was started. Alonso-Sáez et al. (2007) also reported concentrations of heterotrophic bacteria 1–2 orders of magnitude higher than those of picocyanobacteria and autotrophic picoeukaryotes during a monthly sampling carried out in 2003–2004 in the North-Western Mediterranean Sea. In terms of carbon biomass, heterotrophic bacteria are usually less abundant than phytoplankton in coastal waters, although the proportion of bacteria increases with the oligotrophy of the system and its biomass is frequently higher than that of phytoplankton in open oceans (Gasol et al., 1997).

### From the Laboratory to the Environment: Is the *Ostreococcus*–*Roseovarius* Coexistence Prevalent in the Environment?

*Roseovarius* strains have been previously reported to be present in algal cultures, which include *O. tauri* cultures (Abby et al., 2014a). *Roseovarius* sp. MS2 strain commonly grows in cultures of the macroalgae *Ulva mutabilis*, where it takes advantage of the dimethylsulfoniopropionate (DMSP) released by the macroalgae and in turn releases compounds that promote the proper development of the macroalgae (Kessler et al., 2018). A previous 4 day co-culture of *Roseovarius mucosus* strain SMR3 and *Skeletonema marinoi*, a centric diatom, demonstrated that this bacterial strain stimulated the growth rate of the microalga (Johansson et al., 2019).

*Roseobacter*, a group belonging to the same order as *Roseovarius* (i.e., Rhodobacterales), is common in coastal waters and their abundances are correlated with Chl*a* concentrations at a global scale, which could suggest an association with phytoplankton communities (Alonso-Sáez et al., 2007; Wietz et al., 2010; D’Ambrosio et al., 2014). In this regard, it was recently reported that Rhodobacterales usually represented 5–10% of total prokaryotic abundance in surface waters in the Western Mediterranean Sea during mid spring, when phytoplankton bloom occurs (Sebastián et al., 2021).

The global analysis of 313 TARA Ocean metagenomes from 68 stations for taxon co-occurrence based on barcodes from the 18S rDNA and 16S rDNA sequences identified 36 robust associations involving *Ostreococcus* (Lima-Mendez et al., 2015). *Ostreococcus* concentration was positively associated with another eukaryotic taxa 35 times, whereas the only robust co-occurrence with a bacterial taxa was with the genus *Rhodopirellula*. It is important to note that the TARA Ocean sampling sites included mostly open ocean waters and that the corresponding communities sequenced did not contain sequence data affiliated to *O. tauri* but to two divergent sister lineages *O. lucimarinus* and *O. spp* RCC809 (Leconte et al., 2020). So, while the *Roseovarius*–*Ostreococcus* association has not been detected from the metagenomes analyzed in the Lima-Mendez et al. (2015) study, this association may be revealed in future metagenomic studies that include coastal sites, where Mamiellophyceae, which include *Ostreococcus*, have been found to be more prevalent (Tragin and Vaulot, 2018). Alternatively, there may be no need for a taxonomic constraint on mutualistic *Ostreococcus*–Bacteria associations, but rather a metabolic constraint. Indeed, a recent closed microbial community experiment (de Jesús Astacioa et al., 2021) provided evidence of metabolic but not taxonomic constraints on long-term persistence of different heterotrophic bacterial communities with the freshwater green algae *Chlamydomonas reinhardtii*. This metabolic redundancy between taxonomically diverse bacterial lineages may be invoked more generally to explain previous reports of a lack of overlap between bacteria–diatom associations observed in culture collections as opposed to bacteria–diatom associations observed in the natural environment (Crenn et al., 2018).

### Possible Applications of Bacteria for Long-Term Stability of Microalgae Culture

Co-cultivation of microalgae and bacteria may have application for biomass production of microalgae. Indeed, specific bacterial strains may be used to (1) increase algal biomass or (2) limit productivity loss due to contamination by an antagonistic bacterial or (3) lyse the microalgae as part of the harvesting process with the addition of an antagonistic bacteria at the end of the growth phase (Lian et al., 2018). Obviously, these developments require precise knowledge of the interactions between specific microalgae–bacteria pairs (Lian et al., 2018).

As *Ostreococcus* cultures left without subculturing are lost upon 4–5 weeks, the *Ostreococcus* cultures are maintained by subculturing 200 μl in 10 ml fresh sterile L1 culture media in transparent tubes for every 3 weeks. The experimental evidence of the beneficial effect of *Roseovarius* on *O. tauri* RCC4221 is opening promising venues in microalga husbandry as it could decrease the frequency of subculturing and, thus, the risk of contamination by antagonistic bacteria or cross-contamination between strains during the subculturing process.

## Data Availability Statement

The original contributions presented in the study are publicly available. This data can be found here: partial 16S rDNA sequences of these three strains were completed with metagenomic contigs and full length 16S rDNA sequences were submitted to GenBank under accession numbers: OK396682, OK396683, OK396702, and OK396703. Metagenome Assembled Genomes of the microbiome and raw data are available from PRJNA797933.

## Author Contributions

GP planned the experiments. SV performed the co-culture experiments and drafted the first version of the manuscript. SV, MN, AC, and CS performed the cytometry monitoring. MN, AC, FS, and SV were responsible for cultures. FS was responsible for DNA extraction. LI isolated, performed 16S rDNA sequencing, and provided the cultures of bacteria isolated from microcosm. LFB and GP performed the bioinformatic analyses of metagenomes. SV and CC performed the statistical analyses. LFB, CC, and GP wrote the final version. All authors contributed to manuscript editing.

## Conflict of Interest

The authors declare that the research was conducted in the absence of any commercial or financial relationships that could be construed as a potential conflict of interest.

## Publisher’s Note

All claims expressed in this article are solely those of the authors and do not necessarily represent those of their affiliated organizations, or those of the publisher, the editors and the reviewers. Any product that may be evaluated in this article, or claim that may be made by its manufacturer, is not guaranteed or endorsed by the publisher.

## Footnotes

1
https://www.bioinformatics.babraham.ac.uk/projects/trim_galore/

2
https://galaxy.pasteur.fr/

## References

[B1] Abby S. S. Touchon M. De Jode A. Grimsley N. Piganeau G. (2014a). Bacteria in *Ostreococcus tauri* cultures – friends, foes or hitchhikers? *Front. Microbiol.* 5:505. 10.3389/fmicb.2014.00505 25426102 PMC4224133

[B2] Abby S. S. Néron B. Ménager H. Touchon M. Rocha E. P. C. (2014b). MacSyFinder: a program to mine genomes for molecular systems with an application to CRISPR-Cas systems. *PLoS One* 9:e110726. 10.1371/journal.pone.0110726 25330359 PMC4201578

[B3] Abby S. S. Rocha E. P. C. (2017). Identification of protein secretion systems in bacterial genomes using MacSyFinder. *Methods Mol. Biol.* 1615 1–21. 10.1007/978-1-4939-7033-9_1 28667599

[B4] Agogué H. Casamayor E. O. Bourrain M. Obernosterer I. Joux F. Herndl G. J. (2005). A survey on bacteria inhabiting the sea surface microlayer of coastal ecosystems. *FEMS Microbiol. Ecol.* 54 269–280. 10.1016/j.femsec.2005.04.002 16332325

[B5] Alonso-Sáez L. Balagué V. Sà E. L. Sánchez O. González J. M. Pinhassi J. (2007). Seasonality in bacterial diversity in north-west Mediterranean coastal waters: assessment through clone libraries, fingerprinting and FISH. *FEMS Microbiol. Ecol.* 60 98–112. 10.1111/j.1574-6941.2006.00276.x 17250750

[B6] Altschul S. F. Gish W. Miller W. Myers E. W. Lipman D. J. (1990). Basic local alignment search tool. *J. Mol. Biol.* 215 403–410. 2231712 10.1016/S0022-2836(05)80360-2

[B7] Amin S. A. Hmelo L. R. van Tol H. M. Durham B. P. Carlson L. T. Heal K. R. (2015). Interaction and signalling between a cosmopolitan phytoplankton and associated bacteria. *Nature* 522 98–101. 10.1038/nature14488 26017307

[B8] Azam F. Malfatti F. (2007). Microbial structuring of marine ecosystems. *Nat. Rev. Microbiol.* 5 782–791. 10.1038/nrmicro1747 17853906

[B9] Bartling P. Vollmers J. Petersen J. (2018). The first world swimming championships of roseobacters—phylogenomic insights into an exceptional motility phenotype. *Syst. Appl. Microbiol.* 41 544–554. 10.1016/j.syapm.2018.08.012 30224097

[B10] Behringer G. Ochsenkühn M. A. Fei C. Fanning J. Koester J. A. Amin S. A. (2018). Bacterial communities of diatoms display strong conservation across strains and time. *Front. Microbiol.* 9:659. 10.3389/fmicb.2018.00659 29681892 PMC5897529

[B11] Bell W. Mitchell R. (1972). Chemotactic and growth responses of marine bacteria to algal extracellular products. *Biol. Bull.* 143 265–277. 10.2307/1540052

[B12] Blanc-Mathieu R. Sanchez-Ferandin S. Eyre-Walker A. Piganeau G. (2013). Organellar inheritance in the green lineage: insights from *Ostreococcus tauri*. *Genome Biol. Evol.* 5 1503–1511. 10.1093/gbe/evt106 23873918 PMC3762196

[B13] Blanc-Mathieu R. Verhelst B. Derelle E. Rombauts S. Bouget F.-Y. Carré I. (2014). An improved genome of the model marine alga *Ostreococcus tauri* unfolds by assessing Illumina de novo assemblies. *BMC Genomics* 15:1103. 10.1186/1471-2164-15-1103 25494611 PMC4378021

[B14] Boutet E. Lieberherr D. Tognolli M. Schneider M. Bairoch A. (2007). UniProtKB/Swiss-Prot. *Methods Mol. Biol.* 406 89–112. 10.1007/978-1-59745-535-0_4 18287689

[B15] Buchan A. LeCleir G. R. Gulvik C. A. González J. M. (2014). Master recyclers: features and functions of bacteria associated with phytoplankton blooms. *Nat. Rev. Microbiol.* 12 686–698. 10.1038/nrmicro3326 25134618

[B16] Choix F. J. López-Cisneros C. G. Méndez-Acosta H. O. (2018). *Azospirillum brasilense* increases CO_2_ fixation on microalgae *Scenedesmus obliquus*, *Chlorella vulgaris*, and *Chlamydomonas reinhardtii* cultured on high CO_2_ concentrations. *Microb. Ecol.* 76 430–442. 10.1007/s00248-017-1139-z 29327073

[B17] Christie-Oleza J. A. Sousoni D. Lloyd M. Armengaud J. Scanlan D. J. (2017). Nutrient recycling facilitates long-term stability of marine microbial phototroph-heterotroph interactions. *Nat. Microbiol.* 2:17100. 10.1038/nmicrobiol.2017.100 28650444 PMC5495174

[B18] Cooper M. B. Kazamia E. Helliwell K. E. Kudahl U. J. Sayer A. Wheeler G. L. (2019). Cross-exchange of B-vitamins underpins a mutualistic interaction between *Ostreococcus tauri* and *Dinoroseobacter shibae*. *ISME J.* 13 334–345. 10.1038/s41396-018-0274-y 30228381 PMC6331578

[B19] Courties C. Vaquer A. Troussellier M. Lautier J. Chrétiennot-Dinet M. J. Neveux J. (1994). Smallest eukaryotic organism. *Nature* 370 255–255. 10.1038/370255a0

[B20] Crenn K. Duffieux D. Jeanthon C. (2018). Bacterial epibiotic communities of ubiquitous and abundant marine diatoms are distinct in short- and long-term associations. *Front. Microbiol.* 9:2879. 10.3389/fmicb.2018.02879 30564203 PMC6288172

[B21] Croft M. T. Lawrence A. D. Raux-Deery E. Warren M. J. Smith A. G. (2005). Algae acquire vitamin B12 through a symbiotic relationship with bacteria. *Nature* 438 90–93. 10.1038/nature04056 16267554

[B22] D’Ambrosio L. Ziervogel K. MacGregor B. Teske A. Arnosti C. (2014). Composition and enzymatic function of particle-associated and free-living bacteria: a coastal/offshore comparison. *ISME J.* 8 2167–2179. 10.1038/ismej.2014.67 24763371 PMC4992077

[B23] de Jesús Astacioa L. M. Prabhakara K. H. Li Z. Mickalide H. Kuehn S. (2021). Closed microbial communities self-organize to persistently cycle carbon. *Proc. Natl. Acad. Sci. U.S.A.* 118:e2013564118. 10.1073/pnas.2013564118 34740965 PMC8609437

[B24] Denise R. Abby S. S. Rocha E. P. C. (2020). The evolution of protein secretion systems by co-option and tinkering of cellular machineries. *Trends Microbiol.* 28 372–386. 10.1016/j.tim.2020.01.005 32298615

[B25] Fagervold S. K. Urios L. Intertaglia L. Batailler N. Lebaron P. Suzuki M. T. (2013). *Pleionea mediterranea* gen. nov., sp. nov., a gammaproteobacterium isolated from coastal seawater. *Int. J. Syst. Evol. Microbiol.* 63 2700–2705. 10.1099/ijs.0.045575-0 23291888

[B26] Field C. B. Behrenfeld M. J. Randerson J. T. Falkowski P. (1998). Primary production of the biosphere: integrating terrestrial and oceanic components. *Science* 281 237–240. 10.1126/science.281.5374.237 9657713

[B27] Fukami K. Nishijima T. Ishida Y. (1997). Stimulative and inhibitory effects of bacteria on the growth of microalgae. *Hydrobiologia* 358 185–191. 10.1007/978-94-017-2097-7_29

[B28] Gasol J. M. del Giorgio P. A. Duarte C. M. (1997). Biomass distribution in marine planktonic communities. *Limnol. Oceanogr.* 42 1353–1363. 10.4319/lo.1997.42.6.1353

[B29] Grimsley N. Pequin B. Bachy C. Moreau H. Piganeau G. (2010). Cryptic sex in the smallest eukaryotic marine green alga. *Mol. Biol. Evol.* 27 47–54. 10.1093/molbev/msp203 19734297

[B30] Helliwell K. E. Lawrence A. D. Holzer A. Kudahl U. J. Sasso S. Kräutler B. (2016). Cyanobacteria and eukaryotic algae use different chemical variants of vitamin B12. *Curr. Biol.* 26 999–1008. 10.1016/j.cub.2016.02.041 27040778 PMC4850488

[B31] Helliwell K. E. Wheeler G. L. Leptos K. C. Goldstein R. E. Smith A. G. (2011). Insights into the evolution of vitamin B12 auxotrophy from sequenced algal genomes. *Mol. Biol. Evol.* 28 2921–2933. 10.1093/molbev/msr124 21551270

[B32] Ivanova E. P. Christen R. Gorshkova N. M. Zhukova N. V. Kurilenko V. V. Crawford R. J. (2010). *Winogradskyella exilis* sp. nov., isolated from the starfish *Stellaster equestris*, and emended description of the genus *Winogradskyella*. *Int. J. Syst. Evol. Microbiol.* 60 1577–1580. 10.1099/ijs.0.012476-0 19700451

[B33] Johansson O. N. Pinder M. I. M. Ohlsson F. Egardt J. Töpel M. Clarke A. K. (2019). Friends with benefits: exploring the phycosphere of the marine diatom *Skeletonema marinoi*. *Front. Microbiol.* 10:1828. 10.3389/fmicb.2019.01828 31447821 PMC6691348

[B34] Kessler R. W. Weiss A. Kuegler S. Hermes C. Wichard T. (2018). Macroalgal-bacterial interactions: role of dimethylsulfoniopropionate in microbial gardening by *Ulva* (Chlorophyta). *Mol. Ecol.* 27 1808–1819. 10.1111/mec.14472 29290092

[B35] Krasovec M. Eyre-Walker A. Sanchez-Ferandin S. Piganeau G. (2017). Spontaneous mutation rate in the smallest photosynthetic eukaryotes. *Mol. Biol. Evol.* 34 1770–1779. 10.1093/molbev/msx119 28379581 PMC5455958

[B36] Lagesen K. Hallin P. Rødland E. A. Staerfeldt H.-H. Rognes T. Ussery D. W. (2007). RNAmmer: consistent and rapid annotation of ribosomal RNA genes. *Nucleic Acids Res.* 35 3100–3108. 10.1093/nar/gkm160 17452365 PMC1888812

[B37] Lasica A. M. Ksiazek M. Madej M. Potempa J. (2017). The Type IX Secretion System (T9SS): highlights and recent insights into its structure and function. *Front. Cell. Infect. Microbiol.* 7:215. 10.3389/fcimb.2017.00215 28603700 PMC5445135

[B38] Leconte J. Benites L. F. Vannier T. Wincker P. Piganeau G. Jaillon O. (2020). Genome resolved biogeography of Mamiellales. *Genes* 11:66. 10.3390/genes11010066 31936086 PMC7016971

[B39] Li H. Durbin R. (2010). Fast and accurate long-read alignment with Burrows–Wheeler transform. *Bioinformatics* 26 589–595. 10.1093/bioinformatics/btp698 20080505 PMC2828108

[B40] Li H. Handsaker B. Wysoker A. Fennell T. Ruan J. Homer N. (2009). The sequence Alignment/Map format and SAMtools. *Bioinformatics* 25 2078–2079. 10.1093/bioinformatics/btp352 19505943 PMC2723002

[B41] Lian J. Schimmel P. Sanchez-Garcia S. Wijffels R. H. Smidt H. Sipkema D. (2021). Different co-occurring bacteria enhance or decrease the growth of the microalga *Nannochloropsis* sp. CCAP211/78. *Microb. Biotechnol.* 14 1159–1170. 33683803 10.1111/1751-7915.13784 PMC8085966

[B42] Lian J. Wijffels R. H. Smidt H. Sipkema D. (2018). The effect of the algal microbiome on industrial production of microalgae. *Microb. Biotechnol.* 11 806–818. 10.1111/1751-7915.13296 29978601 PMC6116740

[B43] Lima-Mendez G. Faust K. Henry N. Decelle J. Colin S. Carcillo F. (2015). Determinants of community structure in the global plankton interactome. *Science* 348:1262073. 10.1126/science.1262073 25999517

[B44] Lupette J. Lami R. Krasovec M. Grimsley N. H. Moreau H. Piganeau G. (2016). Marinobacter dominates the bacterial community of the *Ostreococcus tauri* phycosphere in culture. *Microb. Symbioses* 7:1414. 10.3389/fmicb.2016.01414 27656176 PMC5013054

[B45] Mansky J. Wang H. Ebert M. Härtig E. Jahn D. Tomasch J. (2022). The influence of genes on the “Killer Plasmid” of *Dinoroseobacter shibae* on its symbiosis with the dinoflagellate *Prorocentrum minimum*. *Front. Microbiol.* 12:804767. 10.3389/fmicb.2021.804767 35154034 PMC8831719

[B46] Marie D. Partensky F. Jacquet S. Vaulot D. (1997). Enumeration and cell cycle analysis of natural populations of marine picoplankton by flow cytometry using the nucleic acid stain SYBR green I. *Appl. Environ. Microbiol.* 63 186–193. 10.1128/aem.63.1.186-193.1997 16535483 PMC1389098

[B47] Marie D. Rigaut-Jalabert F. Vaulot D. (2014). An improved protocol for flow cytometry analysis of phytoplankton cultures and natural samples. *Cytometry A* 85 962–968. 10.1002/cyto.a.22517 25155102

[B48] Mitsutani A. Yamasaki I. Kitaguchi H. Kato J. Ueno S. Ishida Y. (2001). Analysis of algicidal proteins of a diatom-lytic marine bacterium *Pseudoalteromonas* sp. strain A25 by two-dimensional electrophoresis. *Phycologia* 40 286–291.

[B49] Mönnich J. Tebben J. Bergemann J. Case R. Wohlrab S. Harder T. (2020). Niche-based assembly of bacterial consortia on the diatom *Thalassiosira rotula* is stable and reproducible. *ISME J.* 14 1614–1625. 10.1038/s41396-020-0631-5 32203123 PMC7242391

[B50] Muggeo V. M. (2008). Segmented: an R package to fit regression models with broken-line relationships. *R News* 8 20–25.

[B51] Myklestad S. M. (1995). Release of extracellular products by phytoplankton with special emphasis on polysaccharides. *Sci. Total Environ.* 165 155–164.

[B52] Not F. Siano R. Kooistra W. H. C. F. Simon N. Vaulot D. Probert I. (2012). “Chapter one – Diversity and ecology of eukaryotic marine phytoplankton,” in *Advances in Botanical Research. Genomic Insights into the Biology of Algae*, Vol. 64 ed. Piganeau G. (Cambridge, MA: Academic Press), 1–53.

[B53] Nurk S. Meleshko D. Korobeynikov A. Pevzner P. A. (2017). metaSPAdes: a new versatile metagenomic assembler. *Genome Res.* 27 824–834. 10.1101/gr.213959.116 28298430 PMC5411777

[B54] Pinto J. Lami R. Krasovec M. Grimaud R. Urios L. Lupette J. (2021). Features of the opportunistic behaviour of the marine bacterium *Marinobacter algicola* in the microalga *Ostreococcus tauri* phycosphere. *Microorganisms* 9:1777. 10.3390/microorganisms9081777 34442856 PMC8399681

[B55] Quast C. Pruesse E. Yilmaz P. Gerken J. Schweer T. Yarza P. (2013). The SILVA ribosomal RNA gene database project: improved data processing and web-based tools. *Nucleic Acids Res.* 41 D590–D596. 10.1093/nar/gks1219 23193283 PMC3531112

[B56] R Core Team (2021). *R: A Language and Environment for Statistical Computing.* Vienna: R Foundation for Statistical Computing.

[B57] Rambo I. M. Dombrowski N. Constant L. Erdner D. Baker B. J. (2020). Metabolic relationships of uncultured bacteria associated with the microalgae *Gambierdiscus*. *Environ. Microbiol.* 22 1764–1783. 10.1111/1462-2920.14878 31775181

[B58] Rosana A. R. R. Orata F. D. Xu Y. Simkus D. N. Bramucci A. R. Boucher Y. (2016). Draft genome sequences of seven bacterial strains isolated from a polymicrobial culture of coccolith-bearing (C-Type) *Emiliania huxleyi* M217. *Genome Announc.* 4:e00673-16. 10.1128/genomeA.00673-16 27417845 PMC4945805

[B59] Sanchez F. Geffroy S. Norest M. Yau S. Moreau H. Grimsley N. (2019). Simplified transforma? On of *Ostreococcus tauri* using polyethylene glycol. *Genes* 10:399. 10.3390/genes10050399 31130696 PMC6562926

[B60] Sebastián M. Ortega-Retuerta E. Gómez-Consarnau L. Zamanillo M. Álvarez M. Arístegui J. (2021). Environmental gradients and physical barriers drive the basin-wide spatial structuring of Mediterranean Sea and adjacent eastern Atlantic Ocean prokaryotic communities. *Limnol. Oceanogr.* 66 4077–4095. 10.1002/lno.11944

[B61] Seemann T. (2014). Prokka: rapid prokaryotic genome annotation. *Bioinformatics* 30 2068–2069. 10.1093/bioinformatics/btu153 24642063

[B62] Sohn J. H. Lee J.-H. Yi H. Chun J. Bae K. S. Ahn T.-Y. (2004). *Kordia algicida* gen. nov., sp. nov., an algicidal bacterium isolated from red tide. *Int. J. Syst. Evol. Microbiol.* 54 675–680. 10.1099/ijs.0.02689-0 15143006

[B63] Su Y. Yang X. Wang Y. Liu Y. Ren Q. Zhang X.-H. (2017). *Muricauda marina* sp. nov., isolated from marine snow of Yellow Sea. *Int. J. Syst. Evol. Microbiol.* 67 2446–2451. 10.1099/ijsem.0.001992 28741993

[B64] Suminto Hirayama K. (1997). Application of a growth-promoting bacteria for stable mass culture of three marine microalgae. *Hydrobiologia* 358 223–230.

[B65] Tragin M. Vaulot D. (2018). Green microalgae in marine coastal waters: the Ocean Sampling Day (OSD) dataset. *Sci. Rep.* 8:14020. 10.1038/s41598-018-32338-w 30232358 PMC6145878

[B66] Wang X. Li Z. Su J. Tian Y. Ning X. Hong H. (2010). Lysis of a red-tide causing alga, *Alexandrium tamarense*, caused by bacteria from its phycosphere. *Biol. Control* 52 123–130.

[B67] Warren M. J. Raux E. Schubert H. L. Escalante-Semerena J. C. (2002). The biosynthesis of adenosylcobalamin (vitamin B12). *Nat. Prod. Rep.* 19 390–412. 10.1039/b108967f 12195810

[B68] Wickham H. (2011). ggplot2. *WIREs Comput. Stat.* 3 180–185. 10.1002/wics.147

[B69] Wietz M. Gram L. Jørgensen B. Schramm A. (2010). Latitudinal patterns in the abundance of major marine bacterioplankton groups. *Aquat. Microb. Ecol.* 61 179–189. 10.1111/j.1365-294X.2006.03189.x 17284217

[B70] Winnepenninckx B. Backeljau T. De Wachter R. (1993). Extraction of high molecular weight DNA from molluscs. *Trends Genet.* 9:407. 10.1016/0168-9525(93)90102-n 8122306

