# Supplementary figures and images for: Long-Term Stability of Bacterial Associations in a Microcosm of Ostreococcus tauri (Chlorophyta, Mamiellophyceae)
Source: Front Plant Sci. 2022 Apr 8;13:814386. doi: 10.3389/fpls.2022.814386 (PMC9024300; doi:10.3389/fpls.2022.814386)

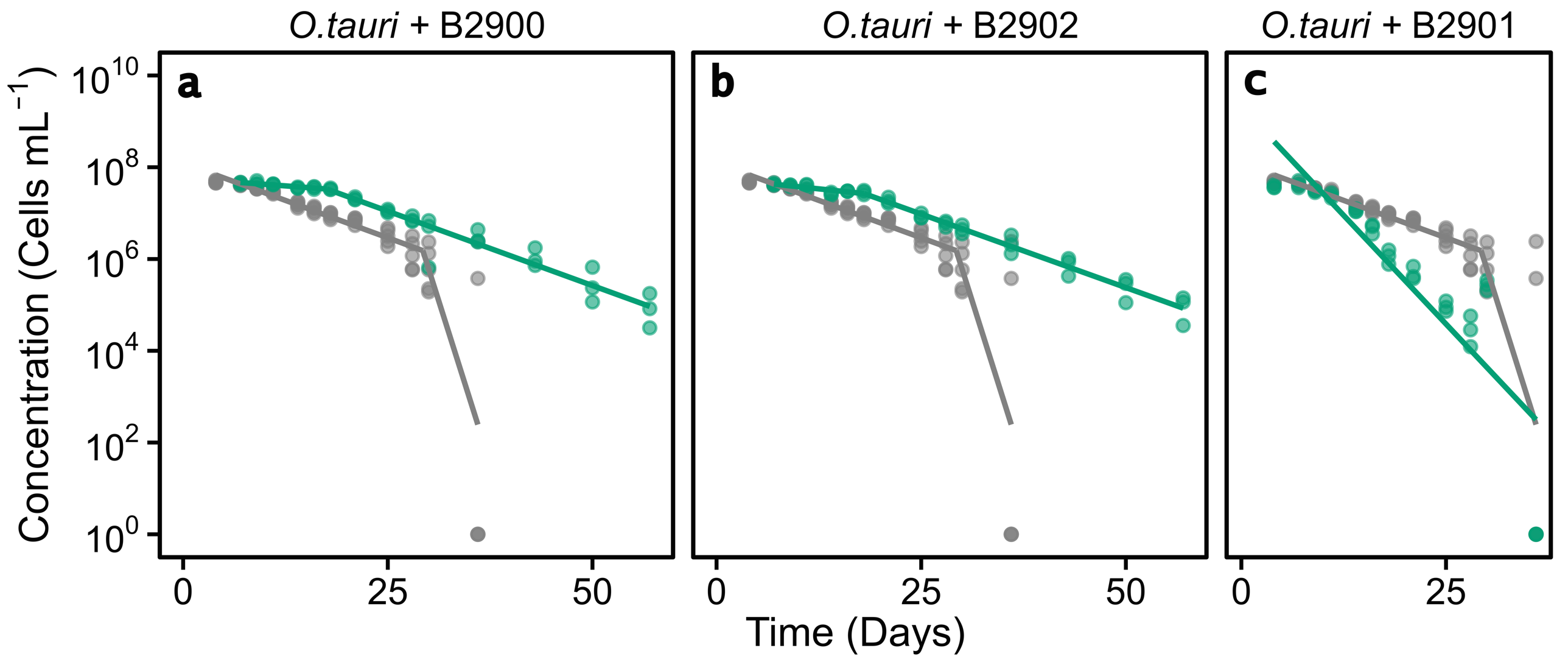

Supplement: Supplementary file 1 [file Image_1.PNG]
